# Extensive Perianal Basal Cell Carcinoma Presenting With Severe Anorectal Dysfunction

**DOI:** 10.14309/crj.0000000000002361

**Published:** 2026-10-09

**Authors:** Sebastian Arambulo-Castillo, Maria Alejandra Gutierrez-Luque, Josh Elefteratos, Lingyu Bao, Grace Kajita, Hilary Hertan

**Affiliations:** 1 Division of General Internal Medicine, Department of Medicine, Albert Einstein School of Medicine, Montefiore Medical Center, Wakefield Campus, Bronx, NY; 2 Facultad de Ciencias de la Salud, Universidad Peruana de Ciencias Aplicadas, Lima, Peru; 3 Division of Gastroenterology, Department of Medicine, Albert Einstein School of Medicine, Montefiore Medical Center, Wakefield Campus, Bronx, NY

**Keywords:** perianal basal cell carcinoma, anorectal dysfunction, rare malignancy

## Abstract

Perianal basal cell carcinoma is an exceedingly rare malignancy that may mimic benign anorectal conditions or anal squamous cell carcinoma. We report an 84-year-old man with a several-year history of a progressively enlarging circumferential perianal lesion, complicated by constipation, chronic fecal incontinence, bleeding, and significant weight loss. Examination revealed a 15 cm ulcerated tumor involving the entire anal verge, with biopsy confirming basal cell carcinoma and sparing of the anal canal mucosa. Imaging showed fecal impaction and perianal soft tissue thickening without metastasis. Given extensive involvement, vismodegib was initiated. This case highlights the importance of early biopsy of atypical perianal lesions.

## INTRODUCTION

Basal cell carcinoma (BCC) is the most common type of skin cancer, accounting for approximately 80% of all nonmelanoma skin malignancies,^1^ and is typically slow-growing, locally invasive, and associated with low metastatic potential.^2^ Although Hedgehog pathway activation is central to BCC pathogenesis, most lesions arise in sun-exposed areas, particularly the head and neck; however, involvement of sun-protected sites such as the perianal and genital regions is exceptionally rare, accounting for less than 0.2% of all BCC cases.^2–4^

Perianal BCC most commonly manifests as a central erythematous ulceration with indurated, raised borders and may overlap with benign anorectal conditions routinely evaluated by gastroenterologist. Reported symptoms include anorectal bleeding (34.3%), pain (30%), and pruritus (18.6%), although some patients may remain asymptomatic.^2^ Management is usually surgical, but extensive disease or high-risk patients may require radiotherapy or systemic Hedgehog pathway inhibition.^1,5^ We present a case of extensive perianal BCC associated with fecal incontinence and intermittent bleeding, highlighting the importance of careful perianal examination and timely biopsy or referral for tissue diagnosis when anorectal symptoms or lesions are persistent, atypical, or progressive.

## CASE REPORT

An 84-year-old man with a remote history of BCC of the right nasal wall and upper eyelid presented after a mechanical fall. During evaluation, he disclosed a longstanding perianal lesion that had progressively enlarged over several years. Over the preceding year, he developed worsening constipation, thin-caliber stools, fecal incontinence, and intermittent bright red blood per rectum. He denied pain or melena and reported unintentional 45-pound weight loss over 6 months.

Physical examination revealed a large circumferential, cauliflower-like lesion involving the entire anal verge and extending approximately 5–7 cm radially, with perianal skin thickening and no active bleeding (Figure 1). Digital rectal examination showed increased sphincter tone and anal canal dilation without gross blood.

Computed tomography (CT) of the abdomen and pelvis showed rectal distention with stool impaction and ill-defined soft tissue thickening at the anal verge extending into the intergluteal cleft, consistent with the perianal lesion. No distant metastatic disease was identified (Figure 2).

Gastroenterology evaluation suggested overflow diarrhea in the setting of chronic fecal incontinence and stool impaction. Enemas and bowel cleansing were deferred because of concern for trauma from the large anal mass.

Examination under anesthesia revealed a circumferential ulcerated mass with rolled edges measuring approximately 15 cm, involving the entire anal verge and extending posteriorly, with partial sparing of the anterior mucosa. Final pathology from biopsies of the left and right perianal skin confirmed BCC, whereas the anal canal biopsy was negative for carcinoma.

Given the advanced symptomatic lesion, age, cognitive concerns, and complex care needs, vismodegib 150 mg daily was initiated. At early follow-up, rectal bleeding resolved and he denied rectal pain, although fecal incontinence persisted. Diverting colostomy was discussed as a palliative option for symptom control, but the patient deferred surgery. At 6-month follow-up, oncology documentation indicated that he had taken vismodegib for 6 weeks, although a later note suggested that treatment may have continued for up to 12 weeks before discontinuation; the exact total duration could not be confirmed. Physical examination showed no obvious recurrent mass. CT pelvis demonstrated interval resolution of the previously described soft tissue mass at the anal verge, with residual irregular soft tissue thickening possibly representing posttreatment change vs residual disease.

**Figure 1.**
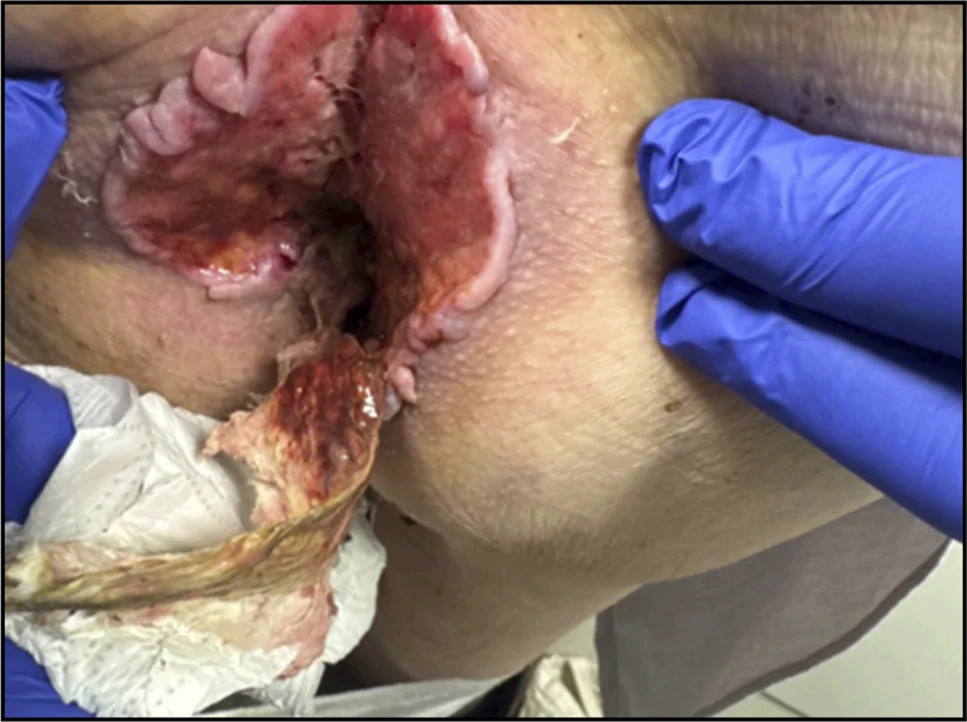
Clinical photograph demonstrating a large circumferential ulcerated perianal lesion involving the entire anal verge, with irregular raised borders and extensive surrounding tissue destruction.

**Figure 2.**
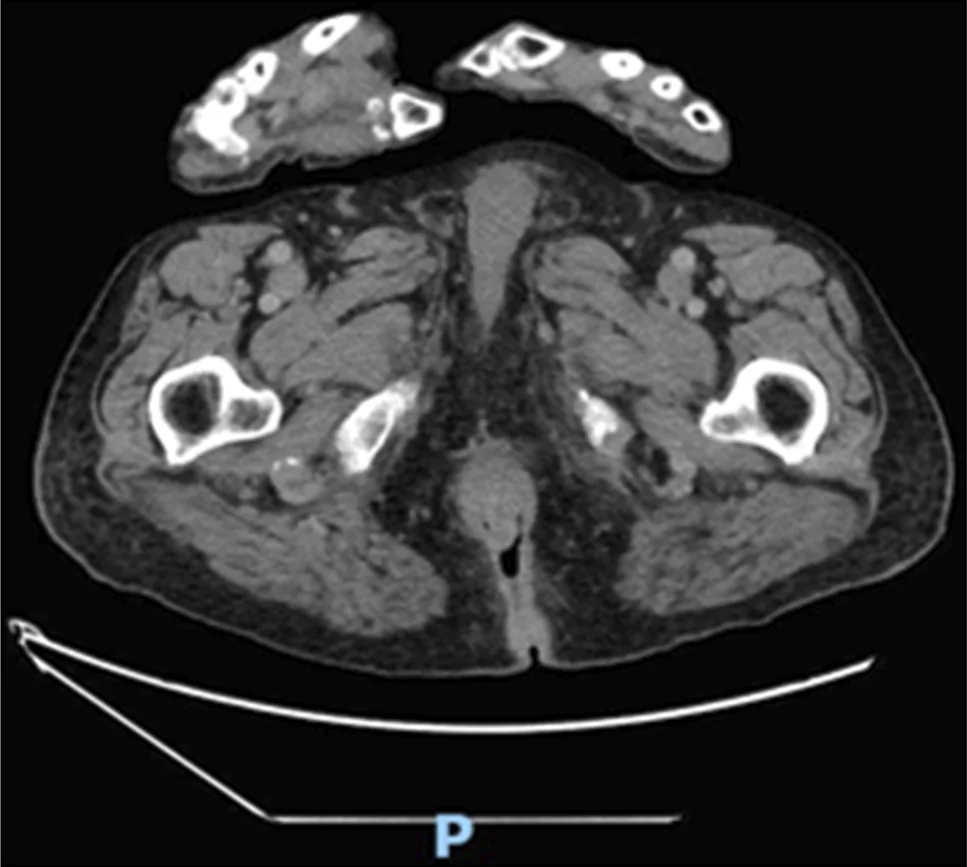
Computed tomography of the abdomen and pelvis with intravenous contrast showing ill-defined soft tissue in the anal region at the verge, with associated foci of air extending into the intergluteal cleft and marked rectal distention due to stool impaction.

## DISCUSSION

The occurrence of BCC in sun-protected sites such as the perianal region suggests that mechanisms beyond ultraviolate radiation (UVR) may contribute to tumor development. Perianal BCC predominantly affects older patients, with a reported median age of 73 years.^6^ In addition, a subset of patients with perianal BCC has a history of nonmelanoma skin cancer at other anatomic sites,^2,6^ raising the possibility of underlying host susceptibility. This highlights the limited understanding of BCC pathogenesis in non-sun-exposed regions.

Previously reported cases of perianal BCC have generally been small, with an average lesion size of 1.95 cm, and most have presented as ulcerated lesions.^6^ Clinically, these tumors may mimic hemorrhoids, anal cysts, or anal squamous cell carcinoma.^7^ Because perianal BCC is typically slow-growing and associated with limited symptom burden, patients may delay seeking medical attention for years.^8,9^

By contrast, our patient's 15 cm circumferential perianal BCC was associated with substantial and anorectal dysfunction, including fecal incontinence, constipation, altered stool caliber, and bleeding. Advanced cases complicated by fecal incontinence and intermittent bleeding have been reported, including an 11 cm lesion; however, presentations with this degree of extent and functional compromise remain exceedingly rare.^10^ Locally aggressive perianal lesions with extension into the anal canal and sphincter complex have been described and may result in marked defecatory dysfunction.^11^

Local excision with negative margins is generally curative for smaller perianal BCC lesions, with no recurrence reported during short-term follow-up of 6–8 months.^5,12^ In poor surgical candidates, radiotherapy has been described as an effective alternative, with clinical improvement reported after 2 months.^8^ For more extensive lesions, palliative surgery followed by radiation has likewise shown favorable outcomes.^10^ In our patient, advanced age, extensive circumferential tumor involvement, cognitive concerns, and complex care needs limited the feasibility of surgery. MRI may have better delineated local tumor extent and possible sphincter involvement; however, MRI was not obtained. CT was used for initial assessment and follow-up, and management was guided by tissue diagnosis, symptom burden, and the patient's decision to defer surgery. Consequently, systemic therapy with a Hedgehog pathway inhibitor was selected. At 6-month follow-up, it showed resolution of rectal bleeding and pain, mild improvement in fecal incontinence, and interval resolution of the anal verge mass on CT, with residual soft tissue thickening possibly representing posttreatment change vs residual disease.

This case emphasizes the gastroenterologist's role in recognizing perianal BCC as a rare malignant cause of chronic anorectal symptoms. Persistent symptoms, including rectal bleeding, altered stool caliber, constipation, fecal incontinence, and weight loss, should prompt careful perianal inspection and consideration of malignancy. Early recognition and biopsy of persistent or atypical perianal lesions may help to prevent progression to functionally compromising disease.

## DISCLOSURES

Author contributions: S. Arambulo-Castillo: conceptualization of the case report; acquisition, analysis, and interpretation of clinical data; drafting and critical revision of the manuscript. MA Gutierrez-Luque: contributed to the final drafting and critical revision of the manuscript. J. Elefteratos: clinical data interpretation; critical revision of the manuscript for important intellectual content. L. Bao: interpretation of diagnostic findings; critical revision of the manuscript. G. Kajita and H. Hertan: supervision; critical revision of the manuscript for intellectual content. All authors gave final approval of the manuscript. S. Arambulo-Castillo is the article guarantor.

Financial disclosure: None to report.

Informed consent was obtained for this case report.

## ABBREVIATIONS:

BCC: Basel cell carcinoma
CT: Computed tomography
MRI: Magnetic resonance imaging
UVR: Ultraviolet radiation
